# Brain structure and intragenic DNA methylation are correlated, and predict executive dysfunction in fragile X premutation females

**DOI:** 10.1038/tp.2016.250

**Published:** 2016-12-13

**Authors:** A L Shelton, K M Cornish, S Kolbe, M Clough, H R Slater, X Li, C M Kraan, Q M Bui, D E Godler, J Fielding

**Affiliations:** 1 School of Psychological Sciences and Monash Institute of Cognitive and Clinical Neurosciences, Faculty of Medicine, Nursing and Health Sciences, Monash University, Melbourne, VIC, Australia; 2 Department of Anatomy and Neuroscience, University of Melbourne, Melbourne, VIC, Australia; 3 Cyto-molecular Diagnostic Research Laboratory, Murdoch Childrens Research Institute and Victorian Clinical Genetics Services, Parkville, VIC, Australia; 4 Department of Paediatrics, University of Melbourne, Melbourne, VIC, Australia; 5 Centre for Epidemiology and Biostatistics, Melbourne School of Population and Global Health, University of Melbourne, Melbourne, VIC, Australia; 6 Department of Medicine, University of Melbourne, Melbourne, VIC, Australia

## Abstract

DNA methylation of the *Fragile X mental retardation 1* (*FMR1*) exon 1/intron 1 boundary has been associated with executive dysfunction in female carriers of a *FMR1* premutation (PM: 55–199 CGG repeats), whereas neuroanatomical changes have been associated with executive dysfunction in PM males. To our knowledge, this study for the first time examined the inter-relationships between executive function, neuroanatomical structure and molecular measures (DNA methylation and *FMR1* mRNA levels in blood) in PM and control (<44 CGG repeats) females. In the PM group, *FMR1* intron 1 methylation was positively associated with executive function and cortical thickness in middle and superior frontal gyri, and left inferior parietal gyrus. By contrast, in the control group, *FMR1* intron 1 methylation was *negatively* associated with cortical thickness of the left middle frontal gyrus and superior frontal gyri. No significant associations were revealed for either group between *FMR1* mRNA and neuroanatomical structure or executive function. In the PM group, the lack of any significant association between *FMR1* mRNA levels and phenotypic measures found in this study suggests that either *FMR1* expression is not well conserved between tissues, or that *FMR1* intron 1 methylation is linked to neuroanatomical and cognitive phenotype in PM females via a different mechanism.

## Introduction

Trinucleotide CGG repeat expansions of the *Fragile X mental retardation 1 (FMR1)* gene are related to a number of Fragile X-associated disorders. Full mutation alleles (FM: greater than 200 CGG repeats) are associated with silencing of *FMR1* through methylation of the promoter region located in the 5′ untranslated region,^1^ resulting in a neurodevelopmental disorder known as Fragile X syndrome. The prevalence of Fragile X syndrome in the general population is ~1 in 4000.^2^ The more common *FMR1* premutation (PM) expansion (55–199 CGG repeats), which is found in ~1 in 209 females and 1 in 430 males,^3^ confers the risk of developing Fragile X-associated tremor/ataxia syndrome (FXTAS). FXTAS is a progressive neurodegenerative disorder, thought to result, in part, from elevated levels of *FMR1* mRNA, leading to protein aggregation (ubiquitin-positive intracellular inclusion bodies likely due to repeat-associated non-AUG-initiated translation) and reduced neuronal cell function.^4, 5, 6^ FXTAS manifests in a range of neurological and clinical symptoms as well as executive dysfunction.^7^ Executive dysfunction, specifically pertaining to working memory and response inhibition processes, has been reported in both PM males^8, 9, 10^ and females without FXTAS,^11, 12, 13, 14, 15^ and may represent either an independent PM phenotype or a precursor to FXTAS.

Significant associations between neuroanatomical structure (white and grey matter) and measures of cognition, including executive function, have been reported in PM males and females.^16, 17, 18, 19, 20, 21^ More recently, a link has also been demonstrated between molecular changes and the risk of developing executive dysfunction in PM females; specifically, methylation changes at the *FMR1* exon 1/intron 1 boundary measured in blood DNA—a region also known as Fragile X-related epigenetic element 2 (FREE2).^22^ To our knowledge, this study for the first time examined whether CGG repeat length, *FMR1* mRNA levels and methylation levels of the CpG island (or the activation ratio, AR) and FREE2 region correlate significantly with altered neuroanatomy in PM females without FXTAS. It also examined the relationships between these molecular and neural measures and cognitive performance; specifically, changes in executive function based on an ocular motor switch task.

## Materials and methods

### Participants

CGG repeat lengths were determined for 36 females aged between 22 and 54 years. Of these, 19 exhibited PM alleles with a CGG repeat length between 55 and 199, and 17 exhibited normal alleles with CGG repeat length <44 (thus providing control data). All were recruited from support groups and population-based Fragile X carrier screening studies,^23^ as well as local networks and via online advertisements.

All participants were English-speaking, had normal (or corrected) vision and hearing, and had no history of any serious neurological damage/disease (including FXTAS). Exclusion criteria extended to those who thought they may be pregnant, as well as those with any magnetic resonance imaging (MRI) contraindication. Ethics approval for this study was granted by the Monash University and Southern Health Human Research Committees (Project Number 10147B); all participants gave their informed consent before inclusion in the study in accordance with the Declaration of Helsinki.

### Molecular analyses

DNA was extracted from whole blood for CGG sizing and methylation analysis. The AmplideX *FMR1* PCR Kit was used for CGG sizing, as per the manufacturer's instructions (Asuragen, Austin, TX, USA). RNA was extracted from peripheral blood mononuclear cells, followed by cDNA synthesis and real-time PCR gene expression analysis performed on a ViiA 7 Real-Time PCR System (Life Technologies, Global). The relative standard curve method was utilised for *FMR1* 5′ and 3′ mRNA quantification normalised to mRNA levels of two internal control genes (*SDHA* and *EIF4A2*), as previously described.^24^ AR was determined using methylation-sensitive Southern blot targeting a *Nru*I restriction site within the *FMR1* CpG island, as previously described.^22^ The EpiTYPER system was used to analyse FREE2 methylation in the blood, consisting of five CpG unit outputs (targeting nine CpG sites) per sample tested.^25^ Blood DNA from each participant was bisulfite-converted in duplicate, with each conversion analysed twice using the EpiTYPER system. A summary measure for each CpG unit was determined as the mean of the four methylation output ratio measurements per sample. These procedures resulted in a total of eight molecular measures: CGG, AR, *FMR1* mRNA, *FMR1* exon 1 (CpG 1 and CpG 2) and intron 1 (CpG 6/7, CpG 8/9 and CpG 10–12) methylation markers.

### Assessment and analysis of executive function

#### Haylings Sentence Completion Test

The Haylings Sentence Completion Test,^26^ a test of response inhibition, required participants to respond to 15 sentences with the last word omitted, by providing a word that was unconnected to the sentence. Responses were classified as either correct, a Category A error (word plausibly finished the sentence) or Category B error (word was somewhat connected to the sentence)—both of which measure inhibitory processing. The total number of Category A and Category B errors were recorded, with larger error numbers indicating impaired response inhibition processes.

#### Ocular motor switch task

The ocular motor switch task^27^ assesses attention, response inhibition and working memory processes. It required participants to move their eye either towards (prosaccade trial) or away (antisaccade) from a target as quickly and as accurately as possible depending on a central colour cue given at the start of each trial (Supplementary Note 1 for more details). As this study was interested in executive dysfunction, antisaccade data were removed from this analysis to avoid any contamination of the paradoxical ‘benefit' that is commonly seen for antisaccade trials following a prosaccade trial (antisaccade switch trials).^28, 29, 30^ This yielded a total of seven prosaccade variables: correct latency (ms), error latency (ms), time to correct (ms), switch/non-switch directional error percentage and switch/non-switch anticipatory error percentage.

### MRI acquisition and analysis

Structural MRIs were acquired on a 3 T Siemens Magneto Skyra scanner using a 20-channel head coil using a T1-weighted three-dimensional MPRAGE scan (208 sagittal slices of 1 mm thickness (no gap), repetition time=1540 ms, echo time=2.55 ms, inversion time=900 ms, a flip angle of 9°, field of view=256 × 256 mm^2^, yielding a standard voxel size=1 × 1 × 1 mm^3^).

T1-weighted three-dimensional MPRAGE data were analysed using FreeSurfer version 5.1.0 (http://surfer.nmr.mgh.harvard.edu) with technical details previously described.^31, 32, 33^ Automated anatomic segmentation procedure was used to measure volume of T1 white matter hypointensities,^32, 34^ whereas regional cortical thickness measures were obtained from the automated anatomic parcellation procedure^34^ for each participant.

Regional cortical thickness from the middle and superior frontal gyri (representing the dorsolateral prefrontal cortex) and inferior parietal gyrus from both left and right hemispheres were selected as they are pivotally involved in the control of saccades.^35, 36, 37^

### Statistical analysis

#### Composite cognitive scores

To reduce the number of executive function variables, separate principal component analyses, using oblique direct rotation with one fixed factor, were hypothesised and tested using the IBM SPSS Statistics software (version 21, IBM, Armonk, NY, USA). This resulted in the creation of three composite cognitive scores: (1) prosaccade response time, (2) prosaccade error score and (3) executive function score (Supplementary Note 2 for more details).

#### Between-group differences

The Stata statistical software (version 14, StataCorp, College Station, TX, USA), was used for all further statistical analyses. Comparisons of demographic information, molecular, composite cognitive scores and neuroanatomical measures between PM and control females were conducted using independent samples *t*-tests (for equal or unequal variances) or Mann–Whitney *U* (when violations of the assumption of normality occurred). The generalised estimating equation was not employed, as correlations within a family were not seen to be significant.

#### Regression models

To assess the inter-relationships between molecular variables, neuroanatomical measures and composite cognitive scores for both PM and control groups, we performed least squares or robust regression analyses (which downweighs the effect of outliers when present). The following models were examined: (I) molecular markers (predictor) and composite cognitive scores (outcome), (II) molecular markers (predictor) and neuroanatomical measures (outcome) and (III) neuroanatomical measures (predictor) and composite cognitive scores (outcome). The goodness of fit was assessed for each regression analysis using the coefficient of determination (*r*^*2*^). Further, the interaction effect of group by (i) composite cognitive score and (ii) neuroanatomical measures was assessed using a general linear model in the IBM SPSS Statistics 21.0. The relationships between *FMR1* mRNA levels and *FMR1* methylation (AR and FREE2 methylation) in both groups were examined using regression analyses.

## Results

### Clinical and molecular intergroup comparisons

PM and control groups were well matched for age, education and full-scale intelligence quotient (assessed via the Wechsler Abbreviated Scale of Intelligence)^38^ (Supplementary Table S1).

**Figure 1:**
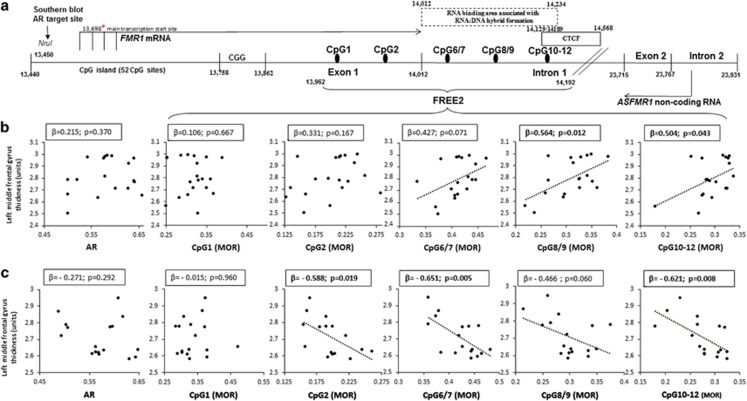
*FMR1* methylation sites and associations with the left middle frontal gyrus in PM and control groups. (**a**) Organisation of the Xq27.3 sequence encompassing specific FREE2 CpG sites (GenBank L29074 L38501) targeted by FREE2 EpiTYPER system. The CTCF box indicates 5′ CTCF-binding sites from UCSF Chip-Seq, which overlap with FREE2 CpG 10-12; the RNA:DNA hybrid box indicates locations of forward and reverse primers used in ChiRP to show formation of RNA:DNA hybrids denoted as fP(200–400) (Colak *et al.*^39^ Figure 4, Supplementary Figure S16 and Supplementary Table S1). Associations between biomarker methylation within *FMR1* CpG island (represented by AR), exon 1 and intron 1 and left middle frontal gyrus thickness (assessed using structural magnetic resonance imaging Model II; unstandardised values) in PM (**b**) and control (**c**) groups. *β* represents standardized coefficients from least or robust (downweighs outliers) regression analysis. AR, activation ratio; *FMR1*, Fragile X mental retardation 1; FREE2, Fragile X-related epigenetic element 2; PM, premutation.

Significant group differences were found for *FMR1* mRNA levels; PM females had a 1.31 mean fold increase in *FMR1* mRNA levels compared with controls (Supplementary Table S1). The mean methylation levels of exon 1 CpG sites 1 and 2; intron 1 CpG sites 6/7, 8/9 and 10–12 and of the CpG island (AR; CpG locations indicated in Figure 1a) were not significantly different between PM and control groups. Further, *FMR1* mRNA levels were not found to be significantly correlated with any *FMR1* methylation measure for either the PM or control group (Supplementary Table S2).

Higher prosaccade error and executive function scores were found for PM females compared with controls, indicating executive dysfunction. No significant differences were found between PM and control groups for prosaccade response time, white matter hypointensities or any cortical thickness measure (Supplementary Table S3).

### Epigenotype–phenotype relationships in PM and control groups

FREE2 methylation levels of *FMR1* intron 1 CpG sites showed the greatest number of significant relationships with composite cognitive scores in the PM group compared with CGG size, AR, exon 1 methylation or *FMR1* mRNA levels in the blood (Table 1). Significant molecular–composite cognitive score relationships were completely absent from the control group (Table 2).

**Figure 2:**
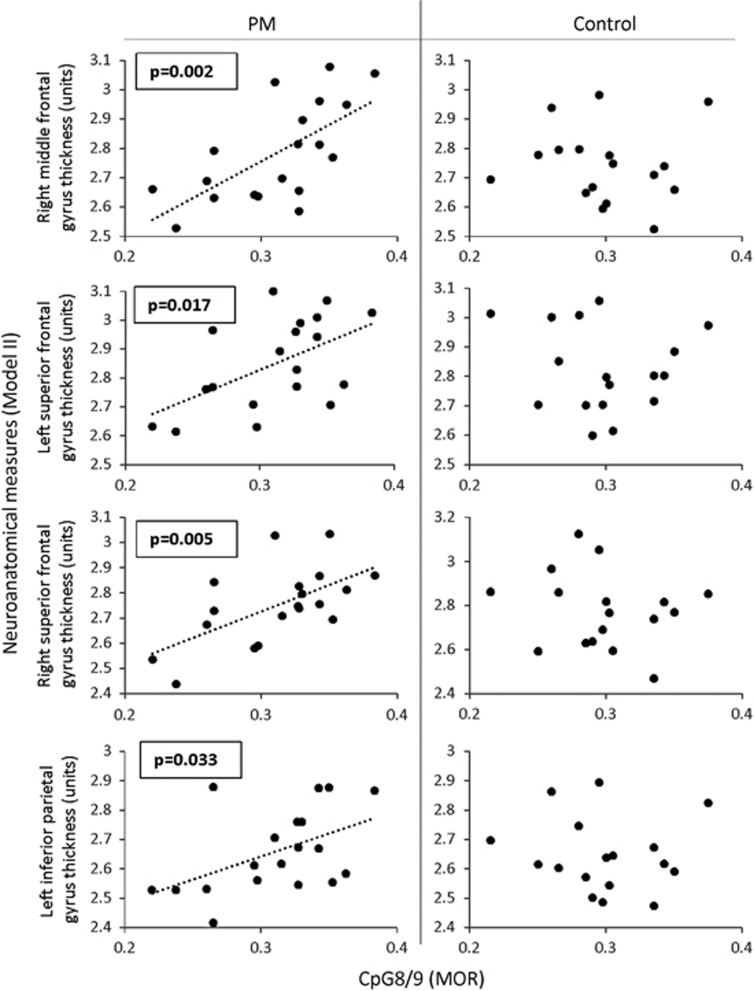
Associations between neuroanatomical cortical thickness and CpG 8/9 methylation in PM and control groups. *P*-values represent results from individual least squares regression analyses assessing the relationship between CpG 8/9 and cortical thickness (unstandardised values), and are presented only for values reaching significance of *P*<0.05. PM, premutation.

Again, *FMR1* intron 1 methylation levels showed the greatest number of significant relationships with neuroanatomical measures for both the PM and control groups compared with CGG size, AR, exon 1 methylation or *FMR1* mRNA levels in the blood (Figure 1 and Table 1). Methylation of *FMR1* intron 1 CpG sites correlated positively with MRI measures in the PM group (Figure 1b, Figure 2 and Table 1). Conversely, for controls, increased methylation of *FMR1* CpG 2, 6/7, 10–12 was associated with decreased cortical thickness in frontal lobe regions. No significant CpG 8/9–neuroanatomical relationships were found for the control group (Figure 1c, Figure 2 and Table 2). Interaction analysis revealed that significant group differences in the relationships between *FMR1* intron 1 methylation and middle frontal, superior frontal and inferior parietal thickness were evident (Table 3).

Neuroanatomical measures were related to executive function measures for both PM and control groups. The three significant relationships for the PM group suggest that executive function deficits, denoted by composite cognitive scores, were related to increased white matter hypointensities (prosaccade response time: coefficient (*β*)=0.491, s.e.=0.211, *P*=0.033, *r*^2^=0.241) and decreased cortical volume in the left middle frontal gyrus (prosaccade error score: *β*=−0.495, s.e.=0.211, *P*=0.031, *r*^2^=0.245), and left inferior parietal gyrus (executive function score: *β*=−0.547, s.e.=0.203, *P*=0.015, *r*^2^=0.299). Conversely, increased bilateral inferior parietal gyrus thickness was positively associated with greater prosaccade error scores in controls (left: *β*=0.439, s.e.=0.166, *P*=0.018, *r*^2^=0.319: *β*=0.490, s.e.=0.225, *P*=0.046, *r*^2^=0.240).

## Discussion

Understanding the disorder-specific role of intragenic DNA methylation is critically important,^40, 41^ providing a unique opportunity to investigate gene/environment interactions of clinical significance.^42^ In this study, highly significant relationships were found between the intragenic methylation within the 5′ end of the *FMR1* intron 1 and phenotype measures of executive function, volume of white matter hypointensities and regional cortical thickness in the frontal and parietal cortices of PM females without FXTAS. The differences in the relationships between methylation markers CpG 6/7 and CpG 8/9 and cortical thickness between PM and control females suggest that in normal neurobiology, *FMR1* methylation (potentially X chromosome inactivation (XCI)) is related to thickness of specific cortical regions and volume of white matter hypointensities, which are disrupted in PM females without FXTAS through a currently unknown mechanism that modifies the observed associations.

### *FMR1* intron 1 methylation, but not *FMR1* mRNA, predicts executive dysfunction in PM females

In PM females without FXTAS, decreased methylation of both *FMR1* promotor (AR) and *FMR1* intron 1 regions was found to relate to executive dysfunction. This relationship was absent in controls entirely. Further, the strongest relationships for each composite cognitive score were seen within the 5′ end of *FMR1* intron 1, as compared with methylation of exon 1 or AR. This is consistent with the study by Cornish and colleagues,^22^ supporting the prior hypothesis that methylation of *FMR1* intron 1 CpG sites is a good predictor of deficits within the executive function phenotype of PM and FM females.^22, 43, 44, 45, 46, 47^

Unlike previous ocular motor studies, *FMR1* mRNA levels were not correlated with executive function scores in this cohort of PM females without FXTAS.^48^ Conversely, *FMR1* intron 1 methylation correlated with both executive function and neuroanatomical structure in the PM group. We also found no significant relationships between any methylation measure (AR and FREE2 methylation) and *FMR1* mRNA for PM or control groups. This suggests that, in PM females without FXTAS, *FMR1* intron 1 methylation has clinical significance involving a different mode or pathway of action that does not directly involve overexpression of *FMR1* mRNA.

It is important to note that in this study *FMR1* mRNA was normalised to two control genes (*SDHA* and *EIF4A2*) and not beta-glucuronidase (*GUS*), as in previous observations assessing differing aspects of executive function.^22, 48, 49^ *GUS* is a commonly used reference gene or internal control for transcript quantification with PCR. In a study of FM males where *FMR1* mRNA was normalised to actin B and *GUS*, a positive linear relationship between *FMR1* mRNA and methylation of the *FMR1* promotor region was found,^50^ which was not evident in this study. This difference could have several explanations including that (a) the Brasa *et al.* study^50^ performed correlation analyses for different CpG sites, (b) used FM males as opposed to PM females without FXTAS, (c) had a much smaller sample size of only seven individuals (susceptible to the effects of outliers) or most likely (d) used a different normalisation strategy of *FMR1* mRNA. In relation to the last potential explanation, it is important to note that variability in gene expression of internal control genes has been well documented to have an impact on target gene real-time PCR outputs,^51^ which we have recently shown to apply in PM females without FXTAS.^52^

### *FMR1* intron 1 differently predicts neuroanatomical structure between PM and control groups

Juxtaposing associations were found between increased FREE2 methylation and cortical thickness in our PM and control groups: increased cortical thickness for the PM group and decreased cortical thickness for the control group. This was most evidenced when assessing the FREE2 methylation relationships with cortical thickness of the left middle frontal gyrus, where there was a trend towards increased cortical thickness for the PM group compared with controls (*P*=0.058). The clear dissociation between *FMR1* intron 1 methylation and cortical thickness of the left middle frontal gyrus, as well as other *FMR1* intron 1 methylation–frontal and inferior parietal relationships, between groups, suggests a possible involvement for XCI skewing in regulating the thickness of this region as part of normal biology. This also suggests that *FMR1* intron 1 methylation in peripheral blood is important when considering XCI in neurological disorders without a PM expansion. This is reinforced by the absence of significant associations between methylation of FREE2 CpG 8/9 and cortical thickness in the control group, compared with the highly significant relationships seen for the PM group. Not only does this study show that methylation of *FMR1* intron 1 CpG sites is a useful biomarker of cortical thickness in PM females without FXTAS, but it also opens up the broader possibility that this may be the case for other disorders involving cortical thickness disruption, such as Alzheimer's (PSEN1 mutations),^53^ Parkinson's,^54, 55^ major depressive disorder^56^ and social anxiety disorder.^57^

### Multiple neuroanatomical correlates of executive function found in the PM group

Each of the composite cognitive scores was found to be associated with either regional cortical thickness or volume of white matter hypointensities within the frontoparietal executive processing network (Model III) for PM females without FXTAS, whereas only inferior parietal thickness related to the prosaccade error score in the control group. Specifically, a positive relationship was found between white matter hypointensities and prosaccade reaction time in PM females without FXTAS, which is consistent with the hypothesis that reduced white matter integrity results in increased response times in cognitive tasks generally.^58^

Similarly to our findings of an association between left middle frontal gyrus thickness and prosaccade error scores, decreased cortical thickness in the middle frontal cortex has been linked to executive dysfunction.^59^ Equally, we also reveal that decreased cortical thickness of the left inferior parietal gyrus related to impaired executive function scores in PM females without FXTAS. Collectively, these findings are in direct contrast to a previous Fragile X syndrome study, where increased cortical thickness was associated with poorer performance on multiple domains of the Stanford-Binet Intelligence Scale.^60^ In that study, the Fragile X syndrome findings were hypothesised to reflect inefficient synaptic pruning due to FMRP deficiencies.^60^ As such, other mechanism(s) and pathways discussed below are likely to underlie these neuroanatomical–executive function relationships in PM females without FXTAS.

### Alternative explanations to the observed relationships

The process of XCI, where only one of the two X chromosomes becomes inactivated in females, is complex and relies on a number of factors including DNA methylation, non-coding RNAs and nuclear protein. DNA methylation is an important process in the regulation of XCI and gene expression. DNA hydroxymethylation (5-hydroxymethylcytosine (5hmC)), is thought to be an epigenetic modifier and a possible intermediate product within an active DNA demethylation pathway, potentially having a role in both neurodevelopmental and neurodegenerative diseases/disorders.^61, 62, 63^ In a FXTAS mouse model, 5hmC levels were found to be reduced compared to wild-type littermates, suggesting that for PM individuals, 5hmC may have a neurodegenerative role.^64^ Moreover, non-coding RNAs are most commonly derived from intragenic DNA regions.^65^ Specifically, RNA:DNA hybrids are thought to form at the location of *FMR1* intron 1 CpG sites^39^ and may also have a role in XCI. Further, overexpression of *ASFMR1* and long non-coding RNA have previously been reported in PM individuals,^66^ and have also been associated with parkinsonism and mitochondrial dysfunction.^24^ Future studies should explore the contribution of the aforementioned pathways as alternative explanations for the relationships observed in this study between *FMR1* intron 1 methylation and phenotype measures.

## Conclusion

Overall, understanding how epigenetic changes influence neuroanatomy, executive function and clinical outcomes is highly important for both *FMR1* PM- and FM-related disorders, and broader neurological disorders influenced by abnormal XCI. Although preliminary, this is, to our knowledge, the first study to link *FMR1* intron 1 methylation and neuroanatomical structure in PM and control females. Second, *FMR1* intron 1 methylation produced the greatest number of associations (for both phenotype measures), compared with *FMR1* exon 1 methylation, AR, CGG repeat size and *FMR1* mRNA levels in the blood, confirming our previous observation.^22^ Frontal and parietal cortical thickness, as well as white matter hypointensities, in brain regions that support executive function, also negatively related to composite cognitive scores. Importantly, differences in the relationships between *FMR1* intron 1 methylation and left middle frontal gyrus thickness, and between CpG site 8/9 and frontal and parietal cortical thickness, suggest that XCI skewing in controls may be critical when assessing changes in cortical thickness in females with other neurological diseases. Whereas we provide specific hypotheses regarding the mechanisms underlying such relationships, further confirmatory analysis of the molecular pathways that link *FMR1* intron 1 methylation to neuroanatomical structure and executive dysfunction are needed to support these assertions for the PM neurocognitive phenotype and in normal neurobiology. Importantly, together with our previous studies, the utility of FREE2 methylation, particularly methylation of the 5′ *FMR1* intron 1 region, as a sensitive measure that relates to both neuroanatomical structure and executive dysfunction in PM females without FXTAS, has been now confirmed.

## Figures and Tables

**Table 1:** Relationships between molecular parameters and composite cognitive scores and neuroanatomical measures outcome variables for the PM group

*Outcome variables*	β (r^2^)							
	*CGG*	*AR*	*FMR1 mRNA*	*CpG 1*	*CpG 2*	*CpG 6/7*	*CpG 8/9*	*CpG 10–12*
Prosaccade response time	−0.142 (0.020)	0.450 (0.199)	0.288 (0.093)	−0.105 (0.011)	0.232 (0.054)	**0.791 (0.366)**	0.321 (0.103)	0.281 (0.064)
Prosaccade error score	0.217 (0.047)	**−0.469 (0.350)**	0.038 (0.001)	**0.364 (0.261)**	−0.253 (0.064)	**−0.76 (0.353)**	**−0.591 (0.344)**	**−0.670 (0.478)**
Executive function score	0.229 (0.053)	−0.196 (0.009)	0.132 (0.017)	0.010 (0.000)	0.213 (0.045)	−0.455 (0.198)	**−0.511 (0.261)**	−0.310 (0.086)
White matter hypointensities	−0.079 (0.006)	0.386 (0.158)	0.265 (0.100)	**−0.511 (0.261)**	0.113 (0.013)	**0.471 (0.222)**	0.076 (0.005)	0.405 (0.079)
Left middle frontal gyrus	−0.146 (0.021)	0.215 (0.051)	−0.254 (0.066)	0.106 (0.011)	0.331 (0.109)	0.427 (0.179)	**0.564 (0.319)**	**0.504 (0.220)**
Right middle frontal gyrus	0.035 (0.001)	0.039 (0.002)	−0.346 (0.116)	0.065 (0.004)	0.055 (0.003)	0.250 (0.062)	**0.655 (0.430)**	0.375 (0.117)
Left superior frontal gyrus	−0.024 (0.001)	−0.051 (0.003)	−0.324 (0.104)	0.252 (0.064)	0.292 (0.085)	0.426 (0.176)	**0.541 (0.293)**	0.455 (0.183)
Right superior frontal gyrus	0.030 (0.001)	0.065 (0.005)	−0.186 (0.033)	0.305 (0.093)	0.276 (0.076)	0.293 (0.086)	**0.612 (0.374)**	0.415 (0.168)
Left inferior parietal gyrus	−0.081 (0.007)	−0.200 (0.040)	−0.294 (0.084)	0.058 (0.003)	0.104 (0.011)	**0.558 (0.312)**	**0.494 (0.241)**	0.317 (0.077)
Right inferior parietal gyrus	−0.020 (0.000)	−0.242 (0.056)	−0.350 (0.119)	−0.039 (0.002)	0.052 (0.003)	0.242 (0.045)	0.089 (0.008)	−0.023 (0.000)

Abbreviations: *β*, standardized regression coefficients; AR, activation ratio; *FMR1*, Fragile X mental retardation 1; PM, premutation; *r*^2^, coefficient of determination.

Note: Figures in bold indicate that *P*<0.05.

**Table 2:** Relationships between molecular parameters and composite cognitive scores and neuroanatomical measure outcome variables for the healthy control group

*Outcome variables*	β (r^2^)							
	*CGG*	*AR*	*FMR1 mRNA*	*CpG 1*	*CpG 2*	*CpG 6/7*	*CpG 8/9*	*CpG 10–12*
Prosaccade response time	−0.249 (0.062)	−0.353 (0.125)	0.475 (0.225)	0.148 (0.020)	−0.267 (0.067)	0.087 (0.008)	0.075 (0.006)	−0.054 (0.003)
Prosaccade error score	0.109 (0.018)	−0.011 (0.000)	−0.282 (0.190)	0.032 (0.002)	0.296 (0.159)	0.314 (0.099)	0.153 (0.023)	0.205 (0.042)
Executive function score	0.201 (0.040)	0.248 (0.061)	−0.402 (0.162)	0.152 (0.023)	0.089 (0.009)	0.016 (0.000)	−0.330 (0.109)	−0.164 (0.027)
White matter hypointensities	−0.426 (0.206)	−0.254 (0.074)	0.360 (0.130)	0.105 (0.011)	0.271 (0.071)	0.418 (0.174)	0.429 (0.184)	0.206 (0.042)
Left middle frontal gyrus	0.117 (0.010)	−0.271 (0.074)	−0.158 (0.025)	−0.015 (0.000)	**−0.588 (0.334)**	**−0.651 (0.424)**	−0.466 (0.217)	**−0.621 (0.386)**
Right middle frontal gyrus	0.133 (0.014)	−0.280 (0.078)	−0.084 (0.006)	−0.124 (0.015)	−0.151 (0.021)	−0.293 (0.086)	−0.045 (0.002)	−0.261 (0.068)
Left superior frontal gyrus	−0.454 (0.063)	−0.212 (0.045)	−0.306 (0.093)	0.253 (0.030)	−0.022 (0.000)	−0.378 (0.143)	−0.119 (0.014)	−0.728 (0.302)
Right superior frontal gyrus	0.164 (0.021)	−0.297 (0.088)	−0.259 (0.067)	−0.076 (0.005)	−0.085 (0.007)	**−0.507 (0.257)**	−0.175 (0.031)	−0.521 (0.166)
Left inferior parietal gyrus	0.098 (0.008)	−0.269 (0.072)	−0.293 (0.086)	−0.349 (0.132)	−0.006 (0.000)	−0.426 (0.182)	−0.076 (0.006)	−0.302 (0.079)
Right inferior parietal gyrus	−0.433 (0.056)	−0.052 (0.002)	−0.340 (0.115)	−0.081 (0.005)	0.060 (0.004)	−0.043 (0.002)	−0.310 (0.087)	−0.301 (0.078)

Abbreviations: *β*, standardized regression coefficients; AR, activation ratio; *FMR1*, Fragile X mental retardation 1; *r*^2^, coefficient of determination.

Note: Figures in bold indicate that *P*<0.05.

**Table 3:** The interaction effect of group by composite cognitive score and neuroanatomical measures for each molecular parameter

*Outcome variables*	F (η_p_^*2*^)							
	*CGG*	*AR*	*FMR1 mRNA*	*CpG 1*	*CpG 2*	*CpG 6/7*	*CpG 8/9*	*CpG 10–12*
Prosaccade response time	0.235 (0.014)	3.038 (0.160)	1.418 (0.081)	0.416 (0.025)	0.983 (0.058)	2.535 (0.133)	0.923 (0.053)	0.556 (0.033)
Prosaccade error score	**15.533 (0.485)**	0.218 (0.013)	1.803 (0.101)	0.185 (0.011)	0.691 (0.041)	1.703 (0.094)	2.108 (0.113)	0.855 (0.049)
Executive function score	**6.620 (0.286)**	1.914 (0.107)	1.077 (0.063)	0.017 (0.001)	1.168 (0.068)	1.905 (0.104)	1.309 (0.074)	2.394 (0.127)
White matter hypointensities	0.421 (0.025)	1.905 (0.104)	2.224 (0.122)	2.125 (0.114)	0.595 (0.036)	3.080 (0.157)	1.664 (0.092)	0.580 (0.034)
Left middle frontal gyrus	1.692 (0.093)	1.012 (0.059)	0.386 (0.024)	0.032 (0.002)	3.241 (0.168)	**8.131 (0.330)**	**6.061 (0.269)**	**6.088 (0.270)**
Right middle frontal gyrus	0.374 (0.022)	0.549 (0.033)	1.069 (0.063)	0.253 (0.015)	0.118 (0.007)	1.405 (0.078)	**5.600 (0.253)**	1.803 (0.099)
Left superior frontal gyrus	0.101 (0.006)	0.319 (0.020)	1.294 (0.075)	0.701 (0.041)	1.050 (0.062)	3.033 (0.155)	**3.532 (0.176)**	2.678 (0.140)
Right superior frontal gyrus	0.072 (0.004)	0.767 (0.046)	0.853 (0.051)	0.797 (0.046)	0.896 (0.053)	**3.497 (0.175)**	**4.596 (0.218)**	1.790 (0.098)
Left inferior parietal gyrus	0.035 (0.002)	0.855 (0.051)	1.134 (0.066)	0.835 (0.048)	0.129 (0.008)	**5.031 (0.234)**	2.717 (0.141)	1.604 (0.089)
Right inferior parietal gyrus	0.045 (0.003)	0.477 (0.029)	1.614 (0.092)	0.177 (0.011)	0.059 (0.004)	0.372 (0.022)	0.120 (0.007)	0.303 (0.018)

Abbreviations: *η*_*p*_^2^, partial eta squared; AR, activation ratio; F, F-test statistic; *FMR1*, Fragile X mental retardation 1.

Note: Figures in bold indicate that *P*<0.05.
