# Supplementary material for: Brain structure and intragenic DNA methylation are correlated, and predict executive dysfunction in fragile X premutation females
Source: Transl Psychiatry. 2016 Dec 13;6(12):e984–. doi: 10.1038/tp.2016.250 (PMC5290342; doi:10.1038/tp.2016.250)
Supplement: Supplementary material [file tp2016250x1.docx]

### SUPPLEMENTARY MATERIAL

#### Supplementary Note 1: Ocular Motor Interleaved Task

Participant’s performed an ocular motor interleaved task within the MRI environment. The task required participants to move their eye either towards (prosaccade) or away (antisaccade) from a target as quickly and as accurately as possible depending on a central colour cue given at the start of each trial (Figure 3). Participants completed a total of 96 prosaccade, 96 antisaccade, and 28 null trials (no target – remain looking at white fixation cross) randomised across 4 experimental blocks. The task stimuli were presented on a projection screen at the rear of the scanner by an LCD projector (maximum flux = 1500 lumens; resolution = 1024 × 768; 60 Hz) viewed at a distance of 155cm.

Horizontal displacement of each participant’s right eye was recorded using a MR-compatible video-based SR Research Eyelink 1000 system, with a spatial resolution of 0.01 degrees and a sampling rate of 500 Hz. Customized software written in Matlab was used to examine the eye trace, marking the time of target onset and offset, as well as time and direction of saccade onset. A criterion of >30⁰/s was used to define saccade onset. Trials featuring blinks (at trial onset), or an unstable baseline (a failure to maintain fixation with 2.5° of central fixation) were removed from further analysis. Each trial was then examined for errors in saccade direction, defined as either looking toward the target circle on antisaccade trials, or looking away from the target circle on prosaccade trials. Anticipatory errors/eye movements, defined as saccades occurring within +/- 100ms of the target circle appearing were also identified and counted as anticipatory errors.

All trials were defined by their trial type (either prosaccade or antisaccade), and secondarily as either a switch (where the current trial was preceded by a dissimilar trial occurred; prosaccade preceded by a antisaccade or an antisaccade preceded by an prosaccade) or non-switch (where a repetition of trial type occurred; prosaccade preceded by a prosaccade or an antisaccade preceded by an antisaccade) trial. Antisaccade trials were then removed from further analysis.


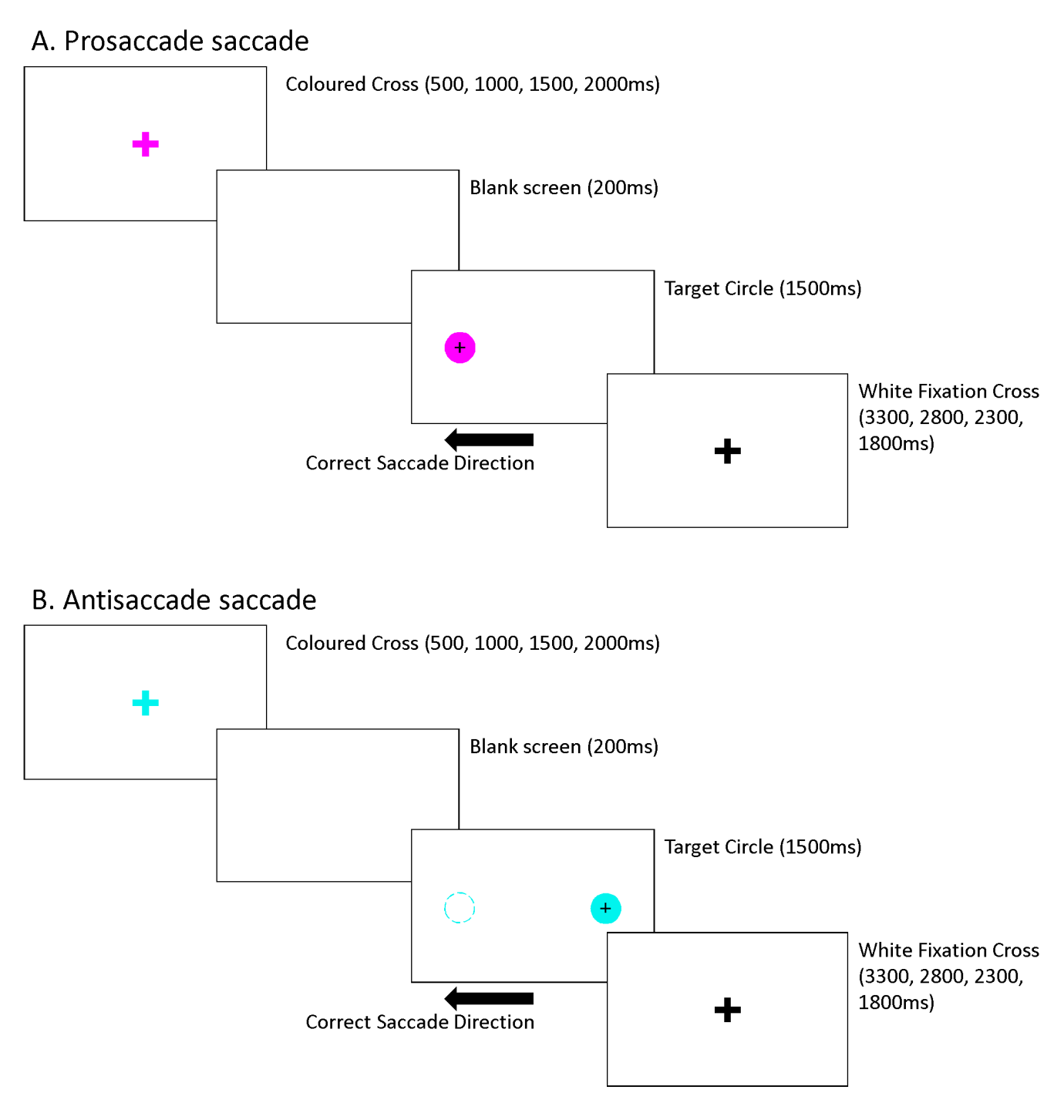
**Figure 3: A schematic diagram of the ocular motor switch task.**

**A)** A prosaccade trial and **B)** An antisaccade trial – the dotted circle indicates the correct response location for an antisaccade trial and does not appear on the screen. Note that each trial lasted a total of 5500ms. The colour indicating the trial type (prosaccade or antisaccade) was counterbalanced between participants.

This yielded a total of seven prosaccade variables: correct latency (ms) (calculated as the difference between target onset and correct saccade onset), error latency (ms) (calculated as the difference between target onset and erroneous saccade onset), time to correct (ms) (calculated as the difference in error saccade completion and corrective saccade onset) (ms), switch/non-switch directional error percentage and switch/non-switch anticipatory error percentage.

#### SUPPLMENTARY NOTE 2: Composite Cognitive Scores

Principle component analysis was used to derive three composite cognitive scores. Here we provide details regarding the variables included in each composite cognitive score and the inter-relationships between the three cognitive measures.

*Prosaccade response time* explained 69.79% of variance in the sample (81.79% of variance in the PM group and 62.68% in the control group), and included prosaccade correct latency, prosaccade error latency and prosaccade time to correct.

*Prosaccade error score* explained 55.77% of variance in the sample (56.72% of variance in the PM group and 38.16% in the control group), and included prosaccade switch/non-switch directional error percentage and prosaccade switch/non-switch anticipatory error percentage.

Finally, *executive function score* explained 50.63% of the variance in the sample (49.28% of variance in the PM group and 45.73% in the control group), and included prosaccade switch/non-switch directional error percentage and Haylings B error score. Inclusion of Haylings A error score in this analysis, explained less than 50% of the variance in the sample, and yielded a non-significant Barlett’s Test of Sphericity (p>0.05), suggesting that its inclusion of Haylings A with the other three variables was inappropriate and unsuitable for principle component analysis.

The three composite scores showed some degree of correlation (*prosaccade response time* vs *executive function score* *r*=-0.557 *p*<0.01; and *prosaccade error score* vs *executive function score r*=0.683 *p<*0.01; *prosaccade response time* vs *prosaccade error score* *r*=-0.307 *p*<0.07).

**Table S1: Statistics on participant demographic and molecular data**

|  | Control (n=17) | | PM (n=19) | | p-value |
| --- | --- | --- | --- | --- | --- |
|  | Mean (SD) | Range | Mean (SD) | Range |  |
| Age (years) | 39.76 (9.72) | 24-54 | 39.37 (9.46) | 22-53 | 0.902 |
| Education (years) | 15.41 (3.02) | 11-26 | 14.84 (3.10) | 9-19 | 0.581 |
| Full Scale IQ | 113.9 (9.22) | 97-130 | 110.95 (9.81) | 88-127 | 0.354 |
| CGG repeat | 30.12 (3.14) | 20-36 | 84.05 (17.0) | 59-123 | **0.000** |
| *FMR1* mRNA | 1.37 (0.37) | 0.90-2.24 | 1.80 (0.62) | 1.03-3.25 | **0.019** |
| Activation ratio | 0.57 (0.05) | 0.49-0.65 | 0.58 (0.05) | 0.50–0.66 | 0.582 |
| CpG 1 | 0.34 (0.50) | 0.29-0.47 | 0.32 (0.04) | 0.25-0.39 | 0.374 |
| CpG 2 | 0.20 (0.03) | 0.16-0.26 | 0.20 (0.04) | 0.14-0.28 | 0.607 |
| CpG 6/7 | 0.42 (0.04) | 0.36-0.48 | 0.41 (0.03) | 0.34-47.0 | 0.392 |
| CpG 8/9 | 0.30 (0.04) | 0.25-0.38 | 0.31 (0.04) | 0.22-0.38 | 0.399 |
| CpG 10-12 | 0.28 (0.04) | 0.18-0.33 | 0.29 (0.04) | 0.18-0.34 | 0.136 |

Note: Figures in bold indicate that p<0.05

**Table S2: Relationships between FMR1 Methylation (predictor) and FMR1 mRNA (outcome) for female premutation carriers and controls.**

|  | PM *FMR1* mRNA | | | Control *FMR1* mRNA | | |
| --- | --- | --- | --- | --- | --- | --- |
|  | β | s.e | p-value | β | s.e | p-value |
| **CpG island (AR)** | -0.082 | 0.278 | 0.771 | -0.207 | 0.253 | 0.425 |
| **CpG 1** | -0.316 | 0.262 | 0.245 | 0.008 | 0.258 | 0.976 |
| **CpG 2** | -0.014 | 0.243 | 0.955 | 0.002 | 0.252 | 0.992 |
| **CpG 6/7** | -0.296 | 0.252 | 0.257 | 0.311 | 0.245 | 0.225 |
| **CpG 8/9** | -0.213 | 0.238 | 0.386 | 0.071 | 0.258 | 0.786 |
| **CpG 10-12** | -0.422 | 0.230 | 0.086 | 0.208 | 0.287 | 0.482 |

Note:β =standardized regression coefficients.

**Table S3: Comparison of white matter hypointensities (volume) and cortical thickness between healthy controls and premutation carriers.**

|  | **Control (n=17)** | **PM (n=19)** | ***p*-value** |
| --- | --- | --- | --- |
|  | Mean (SD) | Mean (SD) |  |
| **White matter hypointensities** | 1306.06 (381.03) | 1492.37 (572.86) | 0.265 |
| **Left middle frontal gyrus** | 2.71 (0.11) | 2.80 (0.16) | 0.058 |
| **Right middle frontal gyrus** | 2.74 (0.13) | 2.78 (0.17) | 0.426 |
| **Left superior frontal gyrus** | 2.82 (0.15) | 2.85 (0.16) | 0.613 |
| **Right superior frontal gyrus** | 2.78 (0.17) | 2.75 (0.15) | 0.591 |
| **Left inferior parietal gyrus** | 2.65 (0.13) | 2.66 (0.15) | 0.757 |
| **Right inferior parietal gyrus** | 2.71 (0.12) | 2.72 (0.13) | 0.718 |
